# Development of a National Caregiver Health Survey for Hematopoietic Stem Cell Transplant: Qualitative Study of Cognitive Interviews and Verbal Probing

**DOI:** 10.2196/17077

**Published:** 2020-01-23

**Authors:** Jacob Kedroske, Sarah Koblick, Dima Chaar, Amanda Mazzoli, Maureen O'Brien, Lilian Yahng, Rebecca Vue, Grant Chappell, Ji Youn Shin, David A Hanauer, Sung Won Choi

**Affiliations:** 1 Blood and Marrow Transplantation Program University of Michigan Ann Arbor, MI United States; 2 School of Public Health University of Michigan Ann Arbor, MI United States; 3 Institute for Social Research Survey Research Operations University of Michigan Ann Arbor, MI United States; 4 Center for Survey Research Indiana University Bloomington, IN United States; 5 College of Communication Arts and Sciences Michigan State University East Lansing, MI United States; 6 Michigan Institute for Clinical and Health Research University of Michigan Ann Arbor, MI United States

**Keywords:** hematopoietic stem cell transplantation, caregivers, mobile applications, qualitative research

## Abstract

**Background:**

Roadmap 1.0 is a mobile health app that was previously developed for caregivers of patients who have undergone hematopoietic stem cell transplantation (HSCT). Formative research targeted toward its end users (caregivers) can help inform app design and development, allowing additional components to be incorporated into the app, which can then be tested in a future randomized controlled trial.

**Objective:**

This study aimed to create a methodologically rigorous national survey that would help inform the development of Roadmap 2.0.

**Methods:**

We conducted a prospective, qualitative research study that took place between November 18, 2018, and February 7, 2019, in a blood and marrow transplant unit within a large academic medical institution in the midwestern part of the United States. Cognitive interviews, including think-aloud and verbal probing techniques, were conducted in 10 adult caregivers (≥18 years) of patients who had undergone HSCT.

**Results:**

Most participants were female (9/10, 90%), white (9/10, 90%), married (9/10, 90%), employed at least part time (6/10, 60%), caregivers of adult patients (7/10, 70%), and had some college education (9/10, 90%) and an annual household income of $60,000 or higher (6/10, 60%). All but one interview was audio-recorded, with permission. Overall, participants were engaged in the cognitive interview process of the draft survey, which included 7 topics. The interviews highlighted areas wherein survey items could be further refined, such as offering more response choices (eg, “NA”) or clarifying the type of transplant (eg, autologous or allogeneic) or context of transplant care (eg, pre-HSCT, during HSCT, post-HSCT, inpatient, and outpatient). Apart from these findings, the items in demographics, caregiving experiences, technology, positive activities, and mood were generally interpreted as intended. On the basis of the transcript data and field notes by the interviewer, items within self-efficacy (Caregiver Self-Efficacy Scale) and coping (Brief Coping Orientation to Problems Experienced inventory) questionnaires generated more confusion among interviewer and participants, reflecting difficulties in interpreting the meaning of some survey items.

**Conclusions:**

This study incorporated the four cognitive aspects of survey methodology that describe the question-answering process—(1) comprehension, (2) information retrieval, (3) judgment and decision making, and (4) responding—by using the think-aloud and probing techniques in cognitive interviews. We conclude that this methodologically rigorous process informed revisions and improved our final questionnaire design.

**International Registered Report Identifier (IRRID):**

RR2-10.2196/resprot.49188

## Introduction

### Background

Millions of individuals depend on family caregivers to manage their care [1]. Although family caregivers are a central part of health care [2], they often are invisible in our health care system, so much so that they are sometimes referred to as “hidden patients” [3]. The economic value of unpaid hours of care by family caregivers was estimated at US $470 billion in 2013, and their contributions continue to intensify [4]. Indeed, with the aging population in the United States and the rising need for caregivers, efforts to foster caregiver health and well-being are essential for sustaining long-term care [5]. Caregivers assist patients with a wide range of activities, including managing complex medical tasks, organizing care plans, and advocating on their behalf [3]. These demands are of a time- and labor-intensive nature, and they place caregivers at high risk for injury and adverse events [3,6-8]. Addressing the needs of at-risk caregivers is an urgent public health priority [1].

Caregiver burden is defined as the “negative reaction to the impact of providing care on the caregiver’s social, occupational, and personal roles” [9]. Much focus has been placed on the wide range of negative implications associated with caregiving [10] (eg, depression and anxiety) [11]. Despite this, most caregivers have recognized the benefits of caregiving [12,13]. The imbalance of focusing primarily on negative aspects may limit our ability to develop new assessment and intervention methods [14]. Thus, a “corrective focus” is needed in caregiving research to expand our knowledge on the positive aspects of caregiving [15,16]. Research on self-management suggests that self-efficacy, a positive aspect, can promote caregiver health, well-being, and positive health behaviors (ie, improved sleep and physical activity) [17,18].

The positive aspects of caregiving may explain how caregivers can positively engage patients in self-care activities [19]. Caregivers with better self-efficacy and well-being (ie, health-related quality of life) may positively affect patients’ health outcomes [20-22]. Simple strategies aimed at enhancing positive thoughts, emotions, and behaviors have been shown to be effective and highly scalable [23-25]. Positive activity interventions, such as daily positive reflection, using gratitude journals, and conducting acts of kindness, have been used in the management of heart disease, cancer, diabetes, and chronic pain [26-29].

Blood and marrow transplant (BMT), commonly referred to as hematopoietic stem cell transplant (HSCT), is an intense but potentially curative therapy for a number of life-threatening blood diseases [30]. Given the high risk associated with BMT, a dedicated caregiver is necessary and expected for at least the first 100 days after the transplant [31]. However, HSCT caregivers are often unprepared for this role; it is not surprising that HSCT caregivers experience significant levels of anxiety and distress, especially during the peritransplant period [32,33]. Psychoeducational, skills training, and therapeutic counseling interventions have been shown to benefit caregiver health and well-being [34]. However, major barriers in translating successful interventions to clinical practice have included (1) limited understanding of the mechanism of action of an intervention and the (2) need for expert trainers, intensive training, and monitoring [3]. Interventions that are mechanism focused, low cost, and sustainable are needed [35].

We recently developed BMT Roadmap (Roadmap 1.0) as a mobile health (mHealth) app to provide patient-specific information, education, and skill-building exercises for caregivers to use during their inpatient stay. The modular components included patient-specific disease characteristics (eg, infectious disease markers, blood type, donor characteristics, and conditioning chemotherapy regimen), laboratory studies (ie, results shown in real time), medications (ie, lists of medications grouped according to indication, eg, antibiotic or antiemetic), clinical trials (ie, easy-to-read description of clinical trials and copies of informed consents), and a health care provider directory (ie, photographs of nurses, physicians, social workers, pharmacists, and nutritionists) in a yearbook style. To date, more than 100 HSCT caregivers have enrolled in institutional review board–approved studies to assess the feasibility of implementing Roadmap 1.0. Major themes that emerged from qualitative interviews conducted with users of Roadmap 1.0 included the following: (1) Roadmap 1.0’s usefulness, ease of use, and likeability; (2) positive aspects of caregiving (ie, benefits of providing care); and (3) desire to expand Roadmap 1.0 to the outpatient setting, specifically targeting “caregiver-specific resources” and “positive activities” components [36-40].

### Objective

Thus, in addition to the qualitative research findings from our single institution, we sought to develop a national caregiver health survey that could be broadly distributed to a diverse sample of HSCT caregivers. The goal of the survey was to examine design considerations for an outpatient version of Roadmap 1.0 (will be referred to as Roadmap 2.0 henceforth). Specifically, our intention was to develop a useful and understandable survey aimed at HSCT caregivers as the target audience. Thus, the aim of this study was to create a methodologically rigorous, broadly national survey that would help further inform the development of the app, in addition to contributing to substantive empirical research on caregivers of patients who have undergone HSCT. To do this, we conducted cognitive interviews to assess each survey item and adjusted, iterated, and rewrote the survey thereafter, which we report herein.

## Methods

### Survey Development Process

**Figure 1:**
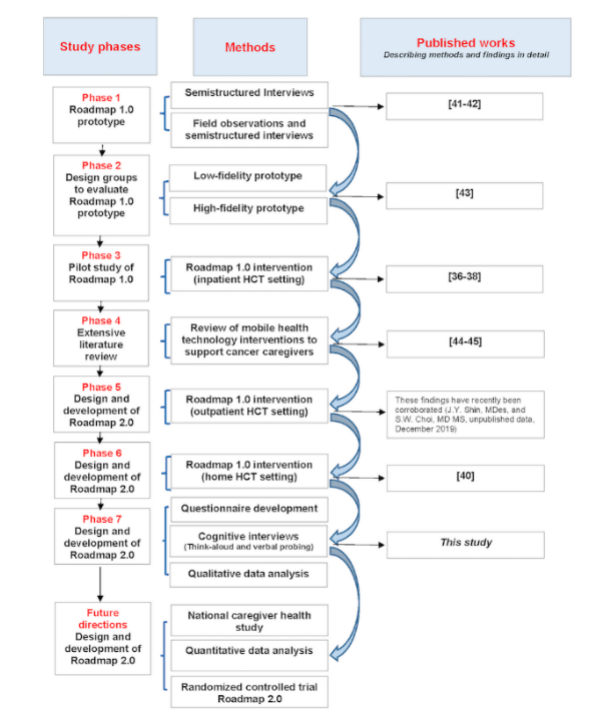
Study phases, methods, and references to published works. HCT: hematopoietic stem cell transplant.

This work is part of a multiphase project (Figure 1) that will develop and test Roadmap 2.0 in a randomized controlled trial.

Development of the Caregiver Health Survey was based on research derived from phases 1 to 6 [36-38,40-45]. The 6 sequential phases led to the development of a draft survey that included (1) demographics, (2) general caregiving duties and life experiences after transplant, (3) use of mobile technology (eg, mHealth apps and wearable sensors), (4) personal enrichment through positive psychology–based activities, (5) mood, (6) confidence in providing care for a loved one (patient) and self-care (self-efficacy), and (7) ability to handle stress and use coping strategies. For items 5 to 7, we incorporated the Patient Health Questionnaire [46], Caregiver Self-Efficacy Scale (CaSES) [47], and Brief Coping Orientation to Problems Experienced (COPE) inventory [48], with permission. The psychometric properties of these instruments are provided in Multimedia Appendix 1.

To ensure that survey items were clearly worded and provided contextualized understanding relevant to HSCT caregiving experiences, we conducted cognitive interviews. In short, the cognitive interview is based on the conceptual frameworks and methods in cognitive and social psychology [49]. As it is an important method used in survey development, particularly under the cognitive aspects of survey methodology (CASM), this approach was used in our survey design to ensure the quality and interpretability of question items [50]. We evaluated interviews using the 4 CASM steps that describe the question-answering process: (1) comprehension, (2) information retrieval, (3) judgment and decision making, and (4) responding [51].

During cognitive interviews, the interviewer read aloud each item and asked the participant to express any questions or concerns regarding the item. The interviewer used both think-aloud interview and verbal probing techniques. Think-aloud interviewing provided an opportunity for open-ended answers without interviewer direction. Verbal probing was used after the participant answered the think-aloud interview to provide further insight into the response [49-51]. In addition, the interviewer recorded field notes or observations from each interview session and also documented handwritten notes after reading each item out loud to the participant.

### Study Recruitment and Informed Consent

The study was approved by the institutional review board (HUM00115569). Cognitive interviews were conducted with family caregivers of patients of the BMT unit of a large academic medical center in the midwestern part of the United States. Eligibility conditions for study participation were that the subject should be (1) the primary family caregiver who had already experienced the transplant procedure with their loved one (patient) and was in the posttransplant phase of care, (2) aged ≥18 years, and (3) comfortable with reading and speaking English. Participants were recruited through referrals from the clinical team (eg, physician or advanced practitioner). The clinical team recruited caregivers who met eligibility criteria from the inpatient and outpatient settings. Only one caregiver declined participation; another caregiver signed the informed consent but was not available on the interview day. Thus, a total of 10 caregivers signed the informed consent and participated in the study.

The cognitive interviews took place between November 19, 2018, and February 7, 2019. Each interview session was approximately 30 to 50 mins in length and was audio-recorded, with permission, and subsequently professionally transcribed (Babbletype LLC). One caregiver participant refused audio-recording. Participants were compensated with a $10 gift card for their participation. A trained project manager with a background in survey methodology (Survey Research Operations, Survey Research Center, Institute of Social Research) moderated the cognitive interviews in a private hospital conference room. The interviewer was not affiliated with the BMT program. Recruitment ended once it was determined that no new data were being identified that informed the content of the survey items. Saturation was defined as a criterion for discontinuing data collection and/or analysis [52].

### Data Analysis

The analysis approach included 3 steps. First, two experts in public health and survey methodology (Survey Research Operations, Survey Research Center, Institute of Social Research), neither affiliated with the BMT program, read the audio-recorded transcripts and the observation and summary notes of each survey item independently. They generated their own notes of each survey item, met together to compare notes, and provided suggested edits (ie, changes to survey items) to the research team. Second, the research team reviewed the results, validated the interpretations and conclusions in a peer-debriefing session, and developed a revised draft survey. Third, a survey methodologist at an external survey research organization (Center for Survey Research) reviewed all of the observation and summary notes and draft survey and provided additional edits of the draft survey. All changes to survey items that led to the final survey were made in collaboration with the lead study investigator.

## Results

### Participant Demographics

As shown in Table 1, the median age of the study participants was 57 years (range: 35-70 years). Most participants were recruited from the inpatient setting (7/10, 70%), female (9/10, 90%), white (9/10, 90%), married (9/10, 90%), caregivers of adult patients who had undergone HSCT (7/10, 70%), and employed at least part time (6/10, 60%) and had some college education (9/10, 90%) and an annual household income of $60,000 or higher (6/10, 60%). Detailed demographics are provided in Multimedia Appendix 2. Overall, participants were engaged in the cognitive interview process. The findings are described below per survey topic. A list of questionnaire items from which the quotes were derived is provided in Multimedia Appendix 3.

**Table 1:** Demographics of the study participants (N=10).

Demographics		Values
Age (years), median (SE); range		52.4 (13.99); 35-70
**Sex, n (%)**		
	Male	2 (20)
	Female	8 (80)
**Race and ethnicity, n (%)**		
	Non-Hispanic white	9 (90)
	Non-Hispanic black	0 (0)
	Hispanic	1 (10)
	Other or multiple	0 (0)
**Marital status, n (%)**		
	Married or in domestic partnership	9 (90)
	Divorced	1 (10)
	Single	0 (0)
**Highest level of schooling, n (%)**		
	High school graduate or general education diploma	1 (10)
	Some college or 2-year college degree	3 (30)
	4-year college graduate	1 (10)
	More than 4-year college degree	5 (50)
**Current employment status, n (%)**		
	Employed part time (up to 39 hours/week)	1 (10)
	Employed full time (40 or more hours/week)	4 (40)
	Self-employed or unable to work	1 (10)
	Homemaker	0 (0)
	Unemployed and not currently looking for work	0 (0)
	Retired	4 (40)
**Annual household income, n (%)**		
	$0-30,001	1 (10)
	$30,001-60,000	2 (20)
	$60,001-100,000	4 (40)
	$100,001-200,000	2 (20)
	Greater than $200,000	1 (10)
	Prefer not to answer	0 (0)

### Survey Topic

#### Demographics

Most caregiver participants were able to respond to demographic items in the survey with ease. A minor finding suggested to include additional response options:

Interviewer: Here’s, “Other (please specify….).” I didn’t see, but still, daughter, that seems like it should have an answer choice.

Participant: Probably pretty common.

Caregiver #06

A survey item asked, “How long ago did the patient receive an allogeneic transplant?” However, caregivers expressed that type of transplant could include autologous (eg, self) or allogeneic (eg, another related donor or another unrelated donor):

Interviewer: Who donated the stem cells for the patient’s transplant? Was it a related donor? An unrelated donor? Or do you know?

Participant: The patient themselves (this refers to autologous transplant).

Caregiver #07

#### Caregiving Experiences

In this section, caregivers expressed that some of the items were not applicable to their caregiving experiences as they were caring for their loved ones (care recipients or patients) at different stages of the transplant. For example, questions about hours spent caregiving did not make sense for a caregiver whose patient was currently hospitalized, undergoing the transplant procedure (ie, care mostly provided by a nurse):

Participant: How long have I been providing care?

Interviewer: Mm-hmm (agreement).

Participant: Thirty-five years, but for this (transplant), a month. Almost, three weeks.

Interviewer: How many hours of caregiving have you provided per week for the patient?

Participant: I’ve probably been here (in the hospital) for 10 hours a day, so 70.

Caregiver #08

#### Technology

Items in this section were considered straightforward and easy to understand by participants. For example, most of the items were quantitative (ie, “How many apps do you use daily?”), and there were no major sources of confusion identified in this section. However, some of the items allowed for only a “Yes” or “No” response, but caregivers preferred a neutral response and suggested a “maybe,” “not applicable (NA),” or “I am not sure” option:

Interviewer: That’s okay, it’s not an answer choice, but I can put that was your first response because that’s not a choice. Which is closer, yes or no, to what you would do?

Participant: I would use it sometimes, so yes.

Caregiver #03

Interviewer: If a caregiver app existed, would you want the app to connect with other caregivers undergoing similar experiences in the transplant experience?

Participant: Sure...I wouldn’t mind texting back and forth. The one-on-one face time I wouldn't necessarily want to do.

Interviewer: Do you feel like maybe you would need a different answer choice like yes, no, or maybe? Participant: Maybe.

Caregiver #10

#### Personal Enrichment Through Positive Activity Exercises

In this section, participants were asked to rate positive psychology activities based on their usefulness and how likely they were to participate in them. Participants were asked to provide a rating for each activity by indicating a response on a scale from 1 (extremely unlikely) to 5 (extremely likely). All of the participants were able to clearly articulate a score. This section was considered straightforward:

Interviewer: Exercise one. I’ll just read through the exercise, and you can tell me on a scale of one to five, how willing you would be able to do the exercise. In exercise one, you would be asked to spend a few minutes each day savoring at least two everyday experiences such as morning coffee, the warmth of the sunshine, a call from a friend. You are to be mindful, very aware of the moment while savoring the experience and using all of your senses, sight, hearing, taste, and touch to solidify the memory. Please rate this activity on a scale of one to five, one, extremely unlikely, two, moderately unlikely, three, neither unlikely or likely, four, moderately likely, five, extremely likely.

Participant: Four.

Interviewer: Four?

Participant: Yes.

Caregiver #03

Interviewer: Exercise two. In this activity, every evening you would think about the things that made you happy that day. You would write down one of these moments on a piece of paper, fold up this piece of paper and drop it into a piggy bank. We would provide the piggy bank. At the end of 30 days, you would close your account, which means you would open the piggy bank and read and savor all of the deposited happy memories. On a scale of one to five, extremely unlikely, moderately unlikely?

Participant: Probably one.

Interviewer: Extremely unlikely.

Caregiver #04

#### Mood

This section included 4 items, which were previously developed in a US sample of 2149 patients from 15 primary care sites [46]. Overall, the items were considered straightforward and easy to follow. Participants seemed to have little to no difficulty following this section’s directions, and they did not express significant concerns about the intent of the items. However, several participants commented that the 4 response options (eg, “not at all,” “several days,” “more than half the days,” and “nearly every day”) were not adequate (ie, a fifth option, such as “every day,” should be included). A participant declined to provide a response because she did not feel comfortable answering some of the items in this section to the interviewer:

Participant: I’m pretty private. I know that seems weird because I’m doing this study.

Interviewer: This is private.

Caregiver #01

#### Confidence in Providing Care to a Loved One (Patient) and Self-Care

The CaSES questionnaire has been previously studied in caregivers of patients with advanced cancer [47]. Participants commented that some of the items in this section were framed with assumptions about the caregivers’ experiences (ie, caregiver of adult vs pediatric patient). Some of the experiences did not apply to all of the participants, depending on the transplant phase (eg, pre-HSCT, during HSCT, or post-HSCT). Most of the caregivers needed clarification on the response options and alluded to needing an “NA” response option:

Interviewer: That’s helpful. Continue to provide care when you feel scared?

Participant: Yes, I can. It’s more about willing and able and definitely will do it, but we haven’t been. Interviewer: You haven’t been scared yet?

Participant: Not yet.

Caregiver 06

Interviewer: How about angry? Continue to provide care when you feel angry?

Participant: That hasn’t happened.

Caregiver #06

#### Ability to Handle Stress and Use Coping Strategies

Participants encountered the most difficulty in interpreting items in this section related to the Brief COPE questionnaire, which has been previously studied in family caregivers of women with advanced breast cancer [48]. Participants reported frustration in responding to questions, such as “I’ve been looking for something good in what is happening” or “I’ve been making fun of the situation,” as they appeared to be insensitive to their journey.

Again, similar to the self-efficacy items, when responding to the coping-related items, acknowledging the caregivers’ frame of reference was important (ie, defining whether the items refer to the pre-HSCT, during HSCT, or post-HSCT setting, or more generally, in the midst of a stressful event). Medical, personal (patient), and family goals also influenced how participants responded to certain items. For example, for some patients, the goal was to work toward more independence. For others, caregivers were instructed to provide as much help as possible to reduce patient suffering:

Interviewer: I’ve been taking action to try and make the situation better.

Participant: That’s the same thing. To me, that question implies a parent could have done something to make it better. It feels like a crappy question. It makes me feel bad like I should have done something differently or I should have taken action to make this better. In reality, parents don’t have control over this.

Caregiver #02

## Discussion

### Principal Findings

In this study, we report the findings of cognitive interviews conducted in caregivers of patients who had undergone HSCT that assessed each survey item. Following the 4 CASM steps that describe the question-answering process—(1) comprehension, (2) information retrieval, (3) judgment and decision making, and (4) responding and using the think-aloud and probing techniques—we found that this methodologically rigorous process informed revisions and improved our final questionnaire design. Indeed, evidence-based data have shown that pilot testing a survey is typically insufficient to ensure the quality and accuracy of the questionnaire [49].

Some of the participants identified confusion within certain sections of the survey that may have been missed with pilot testing alone. Interestingly, the sections that prompted the most concerns were the CaSES and Brief COPE items. For example, the wording of some of items created confusion for our participants, which may have been because of the context of care that is unique to HSCT (eg, pre-HSCT, during HSCT, post-HSCT, inpatient, outpatient, caregiver of an adult patient, or caregiver of a pediatric patient). Thus, this led to the following changes: (1) inclusion of more succinct and clear instructions in the introduction or preamble to each section; (2) incorporation of anchoring terms, such as “at the time of transplant”; and (3) inclusion of questionnaire items tailored to the HSCT population (ie, to better align the items to HSCT, we deleted some items that repeatedly raised concerns in the participants).

Importantly, the interviews revealed that the original set of items was not exhausted and highlighted areas wherein survey items could be further refined by (1) offering more response choices (eg, “NA”), (2) removing some negatively worded items, (3) including more items to make our points clear, (4) moving the order of some items so that it flowed more clearly within each topic, and (5) collapsing redundant items (eg, collapsing income brackets/categories). Thus, we found that during the course of this study, we were able to examine item interpretation and readability and adjust, refine, and rewrite items that would be understood correctly by future survey respondents. We also redesigned the formatting of certain sections by (1) creating a grid or matrix question format with x-axis that listed the item and y-axis that listed the response options, (2) developing a single-response or “radio-question” format, and (3) auto-populating or piping in the patient’s name from a previous response to further reduce the readability burden. Personal enrichment through positive activity exercises did not identify any potential problems that might lead to survey response error, and thus, no further changes were made to any of its items.

### Comparison With Prior Work

Overall, we found that participants were willing to contribute to this type of project, specifically to help future caregivers who would undergo this process, which was consistent with our prior research [53]. Participants of this study were caregivers of both adult and pediatric patients who had undergone BMT. The cognitive interviews identified areas that helped us refine language to allow for interpretability regardless of caregiver type (eg, adult or pediatric). Prior HSCT survey reports have examined either adult or pediatric HSCT [54,55]. Thus, our findings offer a unique contribution to the literature.

Conducting cognitive interviews and using techniques such as think-aloud can help us to learn how participants interpret questionnaire items in their own words, thereby facilitating the development of an instrument that is discriminating, reliable, and valid. de Leeuw et al conducted 2 recent studies [56,57] using a rigorous methodological process that included cognitive interviews in the development of an instructional design evaluation survey. Our findings herein support the use of such rigorous processes in developing surveys that verify how participants are interpreting survey items and whether the survey format and response sets are understandable.

### Strengths and Limitations

Major strengths of the study include the development of a refined survey through rigorous methodology that involved a trained interviewer, experts in qualitative data analysis through the lens of survey methodology (and not affiliated with the BMT study population), a research team with extensive knowledge in the BMT study population, and an external survey methodology research investigator (not affiliated with our institution). Not involving survey methodology experts in our study population helped remove biases in the interpretation of our findings.

On the basis of the review of the transcript data and field notes captured by the moderator, we found that the think-aloud technique was successful in capturing constructive feedback, particularly related to the self-efficacy and coping-related items. Participants freely shared that some items were not pertinent to them, insensitively phrased, or required more response options. Verbal probing revealed items that caused confusion (ie, the participant was stuck or paused for a long time, and the interviewer posed a clarifying question or comment to identify the confusion). Overall, the probes were not directive, and participants were able to verbalize their thoughts freely and openly. In the instances that participants refused to answer an item (ie, because of the insensitive nature of the survey item or privacy concerns), the moderator did not probe further. For example, a caregiver declined to answer the 4 psychological distress items because of privacy concerns. It is possible that survey quality will improve in the future with an anonymous, self-administered survey (ie, removing the interviewer).

Despite our extensive work to develop a survey that would be reliable and interpretable, we recognize the limitations of our work. First, participants who were engaged in this research participated in our research. All but one caregiver agreed to participate when approached by the clinical care team, which may reflect social desirability to please the health care providers. In addition, this could mean that our data were skewed by selective input of those engaged in research. Most participants were white, female, married, and highly educated. We recognize that caregiving experiences could be different based on race, gender, and other identities. Our study was also conducted in a single institution in a midwestern location in the United States. The location could change the needs of a community, and a single-institution study could have reflected the interpretation of individuals attending our center. Although cognitive interviews were conducted to improve questionnaire design and to inform revisions, it is likely that some individuals may still have difficulty interpreting some items as intended, which could lead to inaccurate responses or missing data, if left unanswered.

Nonetheless, findings from our cognitive interviews were invaluable in the refinement of our final Caregiver Health Survey. In general, interventions to support caregivers longitudinally across the trajectory of care are limited. Our larger research agenda aims to contribute to the intervention literature. We hope that the findings from our study, which highlight the importance of cognitive interviews, will be useful for research investigators designing surveys with caregivers in mind, especially surveys in support of developing health interventions. Furthermore, in-depth explorations of survey items by asking caregivers about their perceptions provided them with an important opportunity to include them as active partners in the care of their loved ones (patients). Sharing these data about caregivers’ views of survey items and what their thought processes are when responding to items may also facilitate future caregiving work.

### Future Research

The main goal of this study was to create a survey that will inform the development of our future Roadmap 2.0 app and continue research on mHealth interventions. Data collected herein informed our national caregiver health survey, which was deployed nationally from May to June 2019 (data analyses are forthcoming). Although this investigator-initiated survey queried respondents on health behavior and use of mHealth apps generally to inform our Roadmap 2.0 app, once developed and tested, we anticipate using one of the many recent well-developed and well-validated surveys, such as the Health Information Technology Usability Evaluation Scale [58], mHealth App Usability Questionnaire [59], and/or the Mobile App Rating Scale [60], to assess the app’s usability.

## Appendix

Multimedia Appendix 1 Psychometric properties of the Patient Health Questionnaire-4, Caregiver Self-Efficacy Scale, and Brief Coping Orientation to Problems Experienced inventory.

Multimedia Appendix 2 Detailed demographics of study participants.

Multimedia Appendix 3 List of questionnaire items from which the quotes were derived.

## Abbreviations

BMT: blood and marrow transplant
CaSES: Caregiver Self-Efficacy Scale
COPE: Coping Orientation to Problems Experienced
HSCT: hematopoietic stem cell transplant
mHealth: mobile health
