# Supplementary material for: Development of a National Caregiver Health Survey for Hematopoietic Stem Cell Transplant: Qualitative Study of Cognitive Interviews and Verbal Probing
Source: JMIR Form Res. 2020 Jan 23;4(1):e17077. doi: 10.2196/17077 (PMC7005696; doi:10.2196/17077)
Supplement: Multimedia Appendix 1 [file formative_v4i1e17077_app1.docx]

**Multimedia Appendix 1: Detailed Demographics of the Study Participants**
