# Supplementary material for: Development of a National Caregiver Health Survey for Hematopoietic Stem Cell Transplant: Qualitative Study of Cognitive Interviews and Verbal Probing
Source: JMIR Form Res. 2020 Jan 23;4(1):e17077. doi: 10.2196/17077 (PMC7005696; doi:10.2196/17077)
Supplement: Multimedia Appendix 2 [file formative_v4i1e17077_app2.docx]

**Multimedia Appendix 2: Psychometrics Properties of the PHQ-4, CaSES, and Brief Cope Instruments**

| **PRO** | **DESCRIPTION** | **SCORING** | **PSYCHOMETRICS** |
| --- | --- | --- | --- |
| Patient Health Questionnaire (PHQ-4) | 4-item instrument used to measure psychological distress (depression and anger). | The PHQ–4 begins with the stem question: “Over the last 2 weeks, how often have you been bothered by the following problems?” Responses are scored as 0 (“not at all”), 1 (“several days”), 2 (“more than half the days”), or 3 (“nearly every day”). Therefore, the total score on this composite measure ranges from 0 to 12. | Cronbach's α = 0.85 when measured in family practice and internal medicine sites. |
| Caregiver Self-Efficacy Survey (CaSES) | 28-item, four-factor, instrument used to asses Resilience, Self‐Maintenance, Emotional Connectivity and Instrumental Caregiving. | Each of the four factors has a 4-point response scale with a higher score indicating better functioning. | Cronbach's α = 0.73 - 0.85 when tested with caregivers of people with advanced cancer. |
| Brief Cope | 21-item instrument used to assess 12 different coping strategies: self-distraction, active coping, denial, alcohol/drug use, use of emotional support, behavioral disengagement, venting, positive reframing, planning, use of humor, acceptance, and religion. | The final Symptom Scale consisted of 12 items with higher scores indicating more symptom distress. | Cronbach's α = 0.71 - 0.79 when tested with family caregivers of women with breast cancer. |

Kroenke K, Spitzer RL, Williams JB, Lowe B. An ultra-brief screening scale for anxiety and depression: the PHQ-4. Psychosomatics. 2009 Nov-Dec;50(6):613-21. PMID: 19996233. doi: 10.1176/appi.psy.50.6.613.

Ugalde A, Krishnasamy M, Schofield P. Development of an instrument to measure self-efficacy in caregivers of people with advanced cancer. Psychooncology. 2013 Jun;22(6):1428-34. PMID: 22941754. doi: 10.1002/pon.3160.

Kershaw T, Northouse L, Kritpracha C, Schafenacker A, Mood D. Coping strategies and quality of life in women with advanced breast cancer and their family caregivers. Psychology & health. 2004 Apr;19(2):139-55. PMID: WOS:000220049000001. doi: 10.1080/08870440310001652687.
