# Supplementary material for: Development of a National Caregiver Health Survey for Hematopoietic Stem Cell Transplant: Qualitative Study of Cognitive Interviews and Verbal Probing
Source: JMIR Form Res. 2020 Jan 23;4(1):e17077. doi: 10.2196/17077 (PMC7005696; doi:10.2196/17077)
Supplement: Multimedia Appendix 3 [file formative_v4i1e17077_app3.docx]

Multimedia Appendix 3

List of questionnaire items in which the quotes were derived from:

1. *What is your relationship to the patient (care recipient)? I am the patient’s:*

1. Mother
2. Father
3. Child (18 years and younger)
4. Wife
5. Husband
6. Grandmother
7. Grandfather
8. Sister
9. Brother
10. Aunt
11. Uncle
12. Cousin
13. Neighbor
14. Friend
15. Other (please specific ______)

[CG06] Interviewer: Here’s, “Other (please specify….).” I didn’t see, but still, daughter, that seems like it should have an answer choice. Participant: Probably pretty common.

1. *Who donated stem cells for the patient’s transplant?*

a. Related donor

b. Unrelated donor

c. Do not know

[CG07] Interviewer: Who donated the stem cells for the patient’s transplant? Was it a related donor? An unrelated donor? Or do you know? Participant: The patient themselves (this refers to autologous transplant).

1. *How long did you provide care for the patient?*
   1. 1 month or less
   2. 1.1 – 3 months
   3. 3.1 6 months
   4. 6.1 – 12 months
   5. More than 1 year
   6. Other (please specify)_______

*How many hours of caregiving did you provide per week for the patient? Alternatively, how many hours of caregiving did you provide for the patient in a typical week?*

1. Less than 5 hours
2. 5 – 10 hours
3. 10.1 – 20 hours
4. 20.1 – 30 hours
5. 30.1 – 40 hours
6. More than 40 hours

[CG08] Participant: How long have I been providing care? Interviewer: Mm-hmm (agreement). Participant: Thirty-five years, but for this (transplant), a month. Almost, three weeks. Interviewer: How many hours of caregiving have you provided per week for the patient? Participant: I’ve probably been here (in the hospital) for 10 hours a day, so 70.

1. *As a caregiver, would you use a “caregiver-specific application” that provides information about the patient’s medical experience (e.g., what to expect, graft-versus-host disease, symptoms)?*
   1. Yes
   2. No

[CG03] Interviewer: That’s okay, it’s not an answer choice, but I can put that was your first response because that’s not a choice. Which is closer, yes or no, to what you would do? Participant: I would use it sometimes, so yes.

1. *If a caregiver app existed, would you want the app to connect with other caregivers undergoing similar experiences in the transplant experience?*
   1. Yes
   2. No

[CG10] Interviewer: If a caregiver app existed, would you want the app to connect with other caregivers undergoing similar experiences in the transplant experience? Participant: Sure… I wouldn’t mind texting back and forth. The one-on-one face time I wouldn't necessarily want to do… Interviewer: Do you feel like maybe you would need a different answer choice like yes, no, or maybe? Participant: Maybe.

1. *In this exercise, you would be asked to spend a few minutes each day savoring at least two everyday experiences (e.g., morning coffee, the warmth of sunshine, a call from a friend). You are to be mindful (very aware of the moment), while savoring the experience and use all of your senses (sight, hearing, smell, taste and touch) to solidify the memory.*

*Please rate this activity on a scale of 1 to 5 indicating how willing you are to do this exercise:*

- 1. Extremely unlikely
  2. Moderately unlikely
  3. Neither unlikely nor likely
  4. Moderately likely
  5. Extremely likely

[CG03] Interviewer: Exercise one. I’ll just read through the exercise, and you can tell me on a scale of one to five, how willing you would be able to do the exercise. In exercise one, you would be asked to spend a few minutes each day savoring at least two everyday experiences such as morning coffee, the warmth of the sunshine, a call from a friend. You are to be mindful, very aware of the moment while savoring the experience and using all of your senses, sight, hearing, taste, and touch to solidify the memory. Please rate this activity on a scale of one to five, one, extremely unlikely, two, moderately unlikely, three, neither unlikely or likely, four, moderately likely, five, extremely likely. Participant: Four. Interviewer: Four? Participant: Yes.

1. *In this activity, every evening you would think about the things that made you happy that day. You would write down one of these moments on a piece of paper, fold up this piece of paper and drop it in a piggy bank. (We would provide the piggy bank.) At the end of 30 days, you would “close your account,” which means that you would open the piggy bank and read and savor all the deposited happy memories.*

*Please rate this activity on a scale of 1 to 5 indicating how willing you are to do this exercise:*

- 1. Extremely unlikely
  2. Moderately unlikely
  3. Neither unlikely nor likely
  4. Moderately likely
  5. Extremely likely

[CG04] Interviewer: Exercise two. In this activity, every evening you would think about the things that made you happy that day. You would write down one of these moments on a piece of paper, fold up this piece of paper and drop it into a piggy bank. We would provide the piggy bank. At the end of 30 days, you would close your account, which means you would open the piggy bank and read and savor all of the deposited happy memories. On a scale of one to five, extremely unlikely, moderately unlikely? Participant: Probably one. Interviewer: Extremely unlikely.

1. *Over the last two weeks, how often have you been bothered by:*

*Feeling nervous, anxious or on edge*

- 1. Not at all
  2. Several days
  3. More than half the days
  4. Nearly every day

[CG01] Participant: I’m pretty private. I know that seems weird because I’m doing this study. Interviewer: This is private.

1. *Please rate your level of confidence for the following:*

*Continue to provide care when you feel scared?*

1. I haven’t been doing this at all
2. I’ve been doing this a little bit
3. I’ve been doing this a medium amount
4. I’ve been doing this a lot

[CG06] Interviewer: That’s helpful. Continue to provide care when you feel scared? Participant: Yes, I can. It’s more about willing and able and definitely will do it, but we haven’t been. Interviewer: You haven’t been scared yet? Participant: Not yet.

1. *Please rate your level of confidence for the following:*

*Continue to provide care when you feel angry?*

1. I haven’t been doing this at all
2. I’ve been doing this a little bit
3. I’ve been doing this a medium amount
4. I’ve been doing this a lot

[CG06] Interviewer: How about angry? Continue to provide care when you feel angry? Participant: That hasn’t happened.

1. *I’ve been taking action to try to make the situation better.*
2. I haven’t been doing this at all
3. I’ve been doing this a little bit
4. I’ve been doing this a medium amount
5. I’ve been doing this a lot

[CG02] Interviewer: I’ve been taking action to try and make the situation better. Participant: That’s the same thing. To me, that question implies a parent could have done something to make it better. It feels like a crappy question. It makes me feel bad like I should have done something differently or I should have taken action to make this better. In reality, parents don’t have control over this.
